# Assessment of Renal Risk Score and Histopathological Classification for Prediction of End-Stage Kidney Disease and Factors Associated With Change in eGFR After ANCA-Glomerulonephritis Diagnosis

**DOI:** 10.3389/fimmu.2022.834878

**Published:** 2022-03-22

**Authors:** Benoit Brilland, Charlotte Boud’hors, Marie-Christine Copin, Pierre Jourdain, Nicolas Henry, Samuel Wacrenier, Assia Djema, Clément Samoreau, Jean-Philippe Coindre, Maud Cousin, Jeremie Riou, Anne Croue, Jean-Paul Saint-André, Jean-François Subra, Giorgina Barbara Piccoli, Jean-François Augusto

**Affiliations:** ^1^ Service de Néphrologie-Dialyse-Transplantation, Université d’Angers, Centre Hospitalier Universitaire (CHU) Angers, Angers, France; ^2^ Université d’Angers, Université de Nantes, Centre Hospitalier Universitaire (CHU) d’Angers, Institut National de la Santé Et de la Recherche Médicale (INSERM), Centre National de la Recherche Scientifique (CNRS), Centre de Recherche en Cancérologie et Immunologie Nantes-Angers (CRCI2NA), Structure Fédérative de Recherche Interactions Cellulaires et Applications Thérapeutiques (SFR ICAT), Angers, France; ^3^ Service de Pathologie, Université d’Angers, Centre Hospitalier Universitaire (CHU) Angers, Angers, France; ^4^ Service de Néphrologie-Dialyse, CH de Laval, Laval, France; ^5^ Service de Néphrologie-Dialyse, CH du Mans, Le Mans, France; ^6^ Service de Néphrologie-Dialyse, CH de Cholet, Cholet, France; ^7^ Micro et Nanomedecines Translationnelles, MINT, Université d’Angers, Unité Mixte de Recherche (UMR) Institut National de la Santé Et de la Recherche Médicale (INSERM) 6021, Unité Mixte de Recherche (UMR) Centre National de la Recherche Scientifique (CNRS) 6021, Angers, France; ^8^ Methodology and Biostatistics Department, Delegation to Clinical Research and Innovation, Angers University Hospital, Angers, France

**Keywords:** ANCA, glomerulonephritis, end-stage kidney disease, eGFR, kidney biopsy

## Abstract

**Introduction:**

The “Renal Risk Score” (RRS) and the histopathological classification have been proposed to predict the risk of end-stage kidney disease (ESKD) in ANCA-associated glomerulonephritis (ANCA-GN). Besides, factors associated with kidney function recovery after ANCA-GN onset remain to be more extensively studied. In the present study, we analyzed the value of the RRS and of the histopathological classification for ESKD prediction. Next, we analyzed factors associated with eGFR change within the first 2 years following ANCA-GN diagnosis.

**Materials and Methods:**

We included patients from the Maine–Anjou ANCA-associated vasculitis registry with at least 6 months of follow-up. The values of ANCA-GN, histopathological classification, and RRS, and the factors associated with eGFR variations between ANCA-GN diagnosis and 2 years of follow-up were assessed.

**Results:**

The predictive values of the histopathological classification and RRS were analyzed in 123 patients. After a median follow-up of 42 months, 33.3% patients developed ESKD. The predictive value of RRS for ESKD was greater than that of the histopathological classification. Determinants of eGFR variation were assessed in 80/123 patients with complete eGFR measurement. The median eGFR increased from ANCA-GN diagnosis to month 6 and stabilized thereafter. The only factor associated with eGFR variation in our study was eGFR at ANCA-GN diagnosis, with higher eGFR at diagnosis being associated with eGFR loss (p<0.001).

**Conclusion:**

The RRS has a better predictive value for ESKD than the histopathological classification. The main determinant of eGFR variation at 2 years was eGFR at ANCA-GN diagnosis. Thus, this study suggests that eGFR recovery is poorly predicted by histological damage at ANCA-GN diagnosis.

## Introduction

Anti-neutrophil cytoplasmic antibody (ANCA)-associated vasculitides (AAV) are autoimmune systemic diseases characterized by necrotizing inflammation of small- to medium-sized vessels. Two entities commonly affect kidneys: microscopic polyangiitis, which is associated with myeloperoxidase-ANCA (MPO-ANCA), and granulomatosis with polyangiitis, which is associated with proteinase-3 ANCA (PR3-ANCA) (1–4). Kidney involvement in AAV typically results in crescentic necrotizing glomerulonephritis whose treatment, in addition to steroids, relies on cyclophosphamide or rituximab administration. Despite prompt immunosuppressive treatment, 20%–35% of patients will develop end-stage kidney disease (ESKD) by 5 years following AAV diagnosis, a condition more frequently observed in patients with renal-limited disease and micropolyangeitis as compared to granulomatosis with polyangiitis (5, 6). Meantime, the mortality of AAV patients is driven by infectious complications, cardiovascular diseases, and cancers, all of these complications being promoted by immunosuppressants (7). Thus, it is not surprising that crescentic necrotizing glomerulonephritis, which requires heavy immunosuppressive regimens, has been associated with greater morbidity and mortality in AAV patients (8, 9). The deleterious effects of immunosuppressive drugs are particularly frequent when administered to patients with low glomerular filtration rate (eGFR) at presentation and to elderly patients (10). Whether immunosuppressive treatment minimization or avoidance may represent an option in some subgroups of patients remains an open debate. In this view, patients not recovering after initial dialysis dependence or who develop ESKD early in the course of the disease could be potential candidates.

The identification of patients with ANCA-GN at higher risk of ESKD has been the matter of numerous studies, most of them analyzing the relationship between kidney histology and ESKD risk. In 2010, Berden et al. proposed the histopathological classification dividing patients into four categories based on the proportion of normal, crescentic, and sclerotic glomeruli at kidney biopsy (focal, crescentic, mixed, and sclerotic) (11). This simple classification allowed identifying patient subgroups with different ESKD risk. The focal category has the best prognosis, while the sclerotic one has the worst. However, the crescentic and the mixed categories, representing most ANCA-GN patients, have intermediate and similar prognosis, underlining the need to find new factors that would improve the ESKD classification (12). More recently, Brix et al. proposed a new ESKD risk classification based on the percentage of normal glomeruli, the amount of interstitial fibrosis + tubular atrophy (IFTA), and eGFR at ANCA-GN diagnosis (13). Based on these parameters, a Renal Risk Score (RRS) between 0 and 11 points allows to classify patients in low (0 points), medium (2–7 points), or high (8–11 points) risk categories with respect to risk of ESKD. Using the RRS, the risk of ESKD was 0%, 16%, and 68% in low, medium, and high RRS categories, respectively, in the original cohort (11). However, a substantial fraction of patients classified in the worse subgroups of both ESKD classifications did not evolve towards ESKD, while also patients classified in intermediate risk subgroups reached ESKD (11, 13).

In this context, the identification of factors associated with eGFR increase or decline after ANCA-GN diagnosis could help in improving ESKD prediction. Indeed, it is commonly thought that patients with “active lesions” such as cellular crescents, fibrinoid necrosis, or acute tubular necrosis (ATN) are those with the greatest potential of eGFR gain after ANCA-GN diagnosis (14, 15). However, this question has been only marginally investigated and merits further studies.

Thus, the main objectives of the present study were (1) to analyze the value of the ANCA-GN histopathological classification and RRS categories in our cohort and (2) to analyze factors associated with eGFR variation between ANCA-GN diagnosis and 2 years of follow-up.

## Methods

### Maine–Anjou Registry

The Maine–Anjou registry is a multicenter database that began on January 1, 2018. It includes data from all patients over 18 years old with ANCA-GN diagnosed since January 1, 2000 in the nephrology units of four hospitals (Angers University Hospital and the Regional Hospitals of Le Mans, Cholet, and Laval). Patients included in the registry are at least 18 years old, fulfill the Chapel Hill Consensus Conference criteria for AAV (16), and have presumed or confirmed renal involvement of AAV. The registry collects data concerning presentation at ANCA-GN diagnosis (clinical and biological data), treatment, and outcomes. Data were collected retrospectively at the registry start in 2018 and then updated every 6 months.

The registry has been authorized by the “Commission National Informatique et Liberté” (CNIL, agreement number 2018-MR03-02). In accordance with French law, participants gave their non-opposition to be included in the registry and for the use of their data anonymously. The present study was approved by the local ethic committee of Angers University Hospital (CE 2020/84).

### Study Design

**Figure 1:**
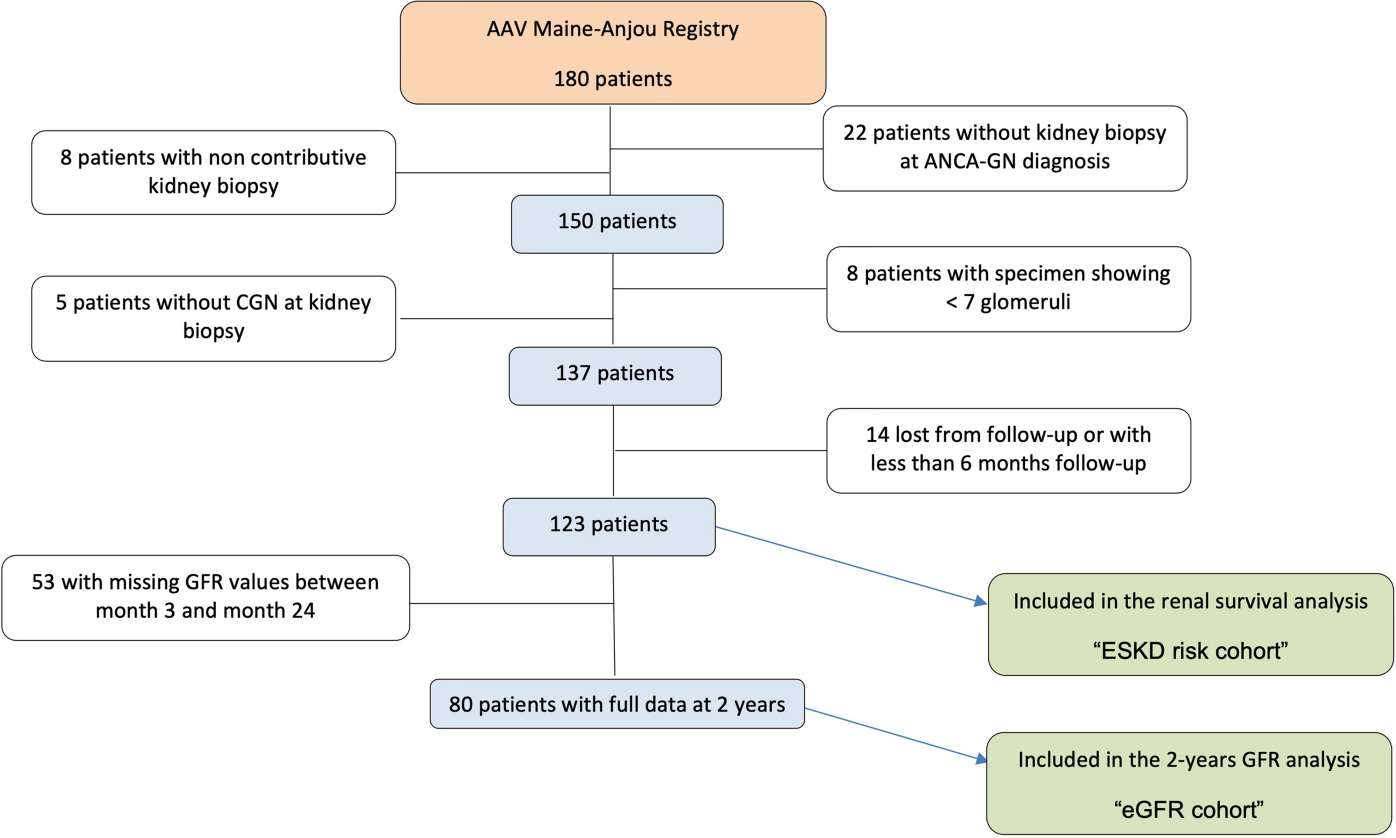
Flowchart of the study.

We considered for inclusion all patients of the registry with new-onset or relapsing ANCA-GN demonstrated by kidney biopsy between January 1, 2000 and January 1, 2020. Patients that were lost to follow-up before 6 months were excluded. Patients with non-contributive biopsy, with less than seven glomeruli, without crescentic pauci-immune glomerulonephritis, and with <6 months of follow-up were also excluded. According to these criteria, 123 patients (“ESKD risk cohort”) were included for the analysis of predictive values of ANCA-GN histopathological classification and of RRS. The analysis of factors associated with eGFR variation between ANCA-GN onset and month 24 was performed in 80 out of these 123 patients from the ESKD cohort with full available eGFR data at ANCA-GN diagnosis at months 3, 6, 12, and 24. This cohort derived from the ESKD cohort was named “eGFR cohort.” Patients with <24 months of follow-up (including patients who died in this period) were excluded from the eGFR cohort (**Figure 1**).

### Data Collection and Definitions

For the present study, the following data were retrieved: age, gender, weight, height, body mass index (BMI), comorbidities (hypertension and diabetes mellitus), and organ involvement at AAV diagnosis. The AAV activity was determined using the Birmingham Vasculitis Activity Score (BVAS) 2003 (17). Medications and biological parameters at AAV diagnosis were retrieved. Patients were followed from ANCA-GN diagnosis until last clinical follow-up or death. The estimated glomerular filtration rate (eGFR) was calculated using the four-variable Modification of Diet in Renal Disease (MDRD) study equation (18). Patients on dialysis were considered to have an eGFR of 5 ml/min/1.73 m^2^. Estimated GFR variation between ANCA-GN onset and month 24 was calculated as follows: (eGFR at 24 months − eGFR at diagnosis). ESKD was defined as the need for renal replacement therapy for more than 3 months or kidney transplantation.

### Renal Histopathology and Calculation of the Renal Risk Score

All kidney biopsies from the four centers are routinely analyzed in a centralized manner in the Pathology Department of the University Hospital of Angers by one of the three renal pathologists (JPSA, AC, or MCC). Kidney biopsy specimens are routinely analyzed using both light microscopy and immunofluorescence. The pathology data are provided in a standardized report, detailing the number and percentage of normal, crescentic, and sclerotic glomeruli. Lesions of fibrinoid necrosis and ATN are also reported qualitatively (presence or absence). All kidney biopsies were specifically analyzed for IFTA quantification by MCC blinded to the patient identification for the purpose of the present study (11). Biopsies with less than seven glomeruli were excluded.

The histopathological classification was determined as described by Berden et al. (11) according to the percentage of sclerotic glomeruli, normal glomeruli, and cellular crescents, in that order. The RRS was calculated as described by Brix et al. (13). Briefly, for each patient, the total RRS was calculated according to the percentage of normal glomeruli (>25%: 0; 10%–25%, 4; and <10%, 6 points), the percentage of IFTA (≤25%, 0 or >25%, 2 points) and the level of eGFR at ANCA-GN diagnosis (>15 ml/min, 0 or ≤15 ml/min, 3 points). According to the total RRS, patients were then classified with low (0 point), medium (2–7 points), and high (8–11 points) risk of ESKD.

### Statistical Analysis

Continuous variables are presented as median and interquartile range. Categorical variables are presented as numbers and percentage. Differences between groups are analyzed using the χ^2^ test (or Fisher’s exact test when applicable) for categorical variables and the Mann–Whitney U test for continuous variables. The Kaplan–Meier method was used to estimate renal survivals. A log-rank test was used to compare the survival curves. To analyze the discrimination capacity of ANCA-GN histopathological classification and RRS for ESKD prediction in the “ESKD risk cohort,” areas under receiver operating characteristic (ROC) curve (AUC) were assessed and further compared using the Delong test (19). Cox analysis was performed to examine factors associated with ESKD in the ESKD cohort. Results are presented as hazard ratio (HR) with 95% CIs. Uni- and multivariable linear regressions were used to analyze factors associated with initial eGFR and eGFR variation between ANCA-GN diagnosis and month 24 in the “eGFR cohort.” Factors with p < 0.1 in the univariable analysis were entered in the multivariable model. All the statistical tests were performed to the two-sided 0.05 level of significance. Statistical analysis was performed using SPSS software^®^ 23.0 and GraphPad Prism^®^.

## Results

### Baseline Data

Among the 180 patients included in the Maine–Anjou AAV registry, 30 were excluded because kidney biopsy was not performed or was not contributive. Five patients had lesions other than ANCA-GN, and eight were excluded because the light microscopy specimen contained less than seven glomeruli. Among the remaining 137 patients, 14 were lost from follow-up before month 6. Thus, the analysis included 123 patients for the renal prognosis analysis (ESKD risk cohort). Among these 123 patients, 80 had complete kidney function data between ANCA-GN onset and month 24 and were considered for the eGFR analysis study (eGFR cohort). The flowchart of the study is presented in **Figure 1**.

Characteristics of the patients in each cohort (“ESKD risk cohort” and “eGFR cohort”) are detailed in **Table 1**. In the ESKD risk cohort, median age at presentation was 69.0 [56.0–74.0] years with a predominance of male sex (61.8%). MPO-ANCAs were detected in 82 (66.7%) patients and PR3-ANCAs in 37 (30.1%), and 4 patients were MPO/PR3 ANCA negative. Median BVAS at ANCA-GN diagnosis was 16.0 [12.0–20.0], and median eGFR at presentation was 17.9 ml/min/1.73 m^2^. Cyclophosphamide was administered to 87.0% of patients as remission–induction regimen. Steroid pulses were used in 102 patients (82.1%) and plasma exchange (PE) in 31 (25.2%). The median follow-up of the cohort was 42.0 [19.2–84.3] months. During follow-up, 41 (33.3%) patients developed ESKD and 36 (29.3%) died. Baseline characteristics of patients included in the eGFR cohort were comparable to those of the ESKD risk cohort (**Table 1**).

**Table 1:** Baseline characteristics of the population and main outcomes.

	ESKD risk cohort	eGFR cohort
	n=123	n=80
**Baseline characteristics**		
Gender, M/F	76/47	53/27
Age, years	69.0 [56.0-74.0]	65.5 [56.0-72.5]
BMI, kg/m^2^	24.6 [22.4-27.9]	24.7 [22.8-28.5]
Hypertension, n (%)	64 (52.0)	37 (46.3)
Diabetes mellitus, n (%)	16 (13.0)	5 (6.3)
**ANCA-associated vasculitis characteristics, n (%)**		
Newly diagnosed	114 (92.7)	78 (97.5)
BVAS at AAV diagnosis or relapse	16.0 [12.0-20.0]	15.5 [12.2-20.0]
ANCA subtype, n (%)		
PR3 ANCA	37 (30.1)	24 (30.0)
MPO ANCA	82 (66.7)	56 (70.0)
ANCA negative	4 (3.3)	–
Organ involvement at onset		
Cutaneous signs, n (%)	22 (18.2)	13 (16.3)
Ear, nose, throat, n (%)	39 (32.0)	28 (35.0)
Heart, n (%)	7 (5.8)	6 (7.5)
Digestive, n (%)	6 (5.0)	4 (5.0)
Lung, n (%)	46 (37.4)	29 (36.3)
Neurological, n (%)	15 (12.4)	7 (8.8)
Renal		
eGFR, ml/min/1.73 m^2^	17.9 [9.6-45.4]	16.8 [6.8-36.3]
Proteinuria, g/g	1.09 [0.56-1.73]	1.22 [0.57-1.84]
Need for renal replacement therapy, n (%)	28 (22.8)	19 (23.8)
**Remission–induction regimen**, n (%)		
Cyclophosphamide	107 (87.0)	74 (92.3)
Rituximab	12 (9.8)	5 (6.3)
Other	4 (3.3)	1 (1.3)
Methylprednisolone pulses	101 (82.1)	69 (86.3)
Plasma exchange	31 (25.2)	22 (27.5)
**Maintenance treatment, n (%)**		
Azathioprine	63 (51.2)	45 (56.3)
Rituximab	36 (29.3)	24 (30.0)
**Outcomes**, n (%)		
End-stage renal disease	41 (33.3)	30 (37.5)
Death	36 (29.3)	18 (22.5)
**Follow-up** (months)	42.0 [19.2-84.3]	58.2 [34.2-98.8]

The ESKD risk cohort included 123 patients and was used to analyze the predictive value of histological classification and RRS for ESKD. The eGFR cohort included 80 out of the 123 patients of the ESKD cohort for whom eGFR was fully available at months 3, 6, 12, and 24 from ANCA-GN diagnosis. Data are presented as median and 25–75 percentiles for continuous variables and absolute value and percentage for categorical variables.

ANCA, anti-neutrophil cytoplasmic antibodies; BMI, body mass index; GN, glomerulonephritis; BVAS, Birmingham Vasculitis Activity Index; MPO, myeloperoxidase; PR3, proteinase-3; eGFR, estimated glomerular filtration rate.

### Pathological Assessment in the ESKD Risk Cohort

**Supplementary Figure S1** reports the renal biopsy findings in the ESKD risk cohort (n=123). The light microscopy specimen contained a median of 20 (13–26) glomeruli and a median proportion of normal, crescentic, and sclerotic glomeruli of 29% [13–47], 43% [29–61], and 12% [5–34], respectively. IFTA involving more than 25% of the biopsy; ATN and fibrinoid necrosis were observed in 42 (34.1%), 88 (71%), and 90 (72.6%) biopsies, respectively. According to Berden histopathological classification, the mixed class was the most represented (n=50, 40.7%) followed by focal (n=29, 23.6%), crescentic (n=25, 20.3%), and sclerotic classes (n=19, 15.4%).

### Renal Risk Score Assessment in the ESKD Risk Cohort

We analyzed patient characteristics according to the RRS as proposed by Brix et al. (13). As expected, kidney involvement (baseline eGFR, proteinuria, and frequency of renal replacement therapy at ANCA-GN diagnosis) was less severe in low as compared to higher risk categories. As also expected, ESKD and death rate were significantly higher in medium and high RRS groups as compared to low RRS group. These data are reported in **Supplementary Table S1**. As for the distribution of each component of the RRS in our cohort, high RRS was associated with a higher proportion of patients with low percentage of normal glomeruli, higher IFTA, and lower eGFR as compared to low RRS. These data are presented in **Supplementary Figure S2**.

### Prognostic Value of the Histopathological Classification and of the Renal Risk Score for ESKD in the ESKD Risk Cohort

**Figure 2:**
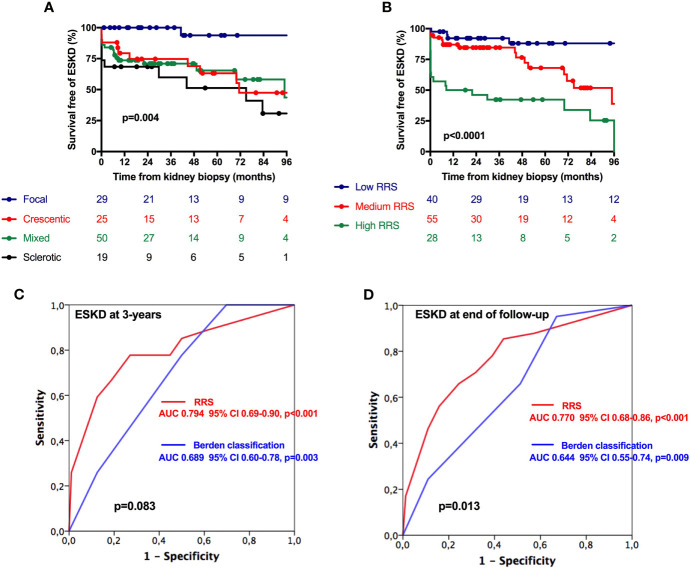
Renal survival in the ESKD cohort according to Berden classification and Renal risk score. **(A)** Survival free of ESKD according to Berden classification and **(B)** Renal Risk Score. **(C, D)** Area under the curve of Renal Risk Score (red line) and Berden classification (green line) for ESKD prediction at 3 years **(C)** and at the end of follow-up **(D)**. Survival curves were performed using Kaplan–Meir method, and comparisons between curves were done using the log-rank test. Black p-values indicate AUC comparisons of Berden classification and RRS. AUC, area under the curve; RRS, renal risk score.

We next analyzed renal survival according to histopathological classification and RRS categories (**Figure 2**). As shown in **Figure 2**, the focal class was associated with the best renal survival, while sclerotic class had the worse prognosis (**Figure 2A**). The estimated renal survival at 5 years of the focal, crescentic, mixed, and sclerotic classes was 93.8%, 63.2%, 65.5%, and 51.3%, respectively. Using RRS, as expected, low-risk patients had the better prognosis and high-risk category the worse one (**Figure 2B**). The estimated renal survival at 3 years in the low, medium, and high-risk categories was 92.2%, 84.6%, and 42.3%, respectively. The area under the curve for ESKD prediction at the end of follow-up of histopathological classification and RRS were 0.644 (p=0.009) and 0.770 (p<0.001), respectively (**Figure 2D**). The predictive value of RRS for ESKD was not statistically different than those of the histopathological classification at 3 years of follow-up (p=0.083, **Figure 2C**) but was significantly higher when considering the end of follow-up (p=0.013, **Figure 2D**).

According to RRS, we observed an increased risk of ESKD in patients classified in the medium- and high-risk categories with respect to the low-risk category (**Supplementary Figure S3**). When components of the RRS were separately analyzed, the percentages of normal glomeruli and eGFR at baseline were significantly associated with ESKD risk. However, we did not observe any significant association between IFTA and ESKD risk. Using ROC curve analysis, we did not observe any significant difference in ESKD prediction at 3 years and at end of follow-up between RRS score and a modified RRS excluding IFTA (**Supplementary Figure S4**).

**Supplementary Figure S5** reports the incidence of ESKD according to RRS score at 3 years and at the end of follow-up in the cohort. Interestingly, among the eight patients with the highest RRS, seven required renal replacement therapy at ANCA-GN diagnosis. Two of them recovered renal function early after ANCA-GN diagnosis but then reached ESKD at 3 months for the first one and 2.5 years for the second one. One patient was still free from renal replacement therapy at 3.5 years from ANCA-GN diagnosis.

### Analysis of Factors Associated With eGFR Variation Between ANCA-GN Diagnosis and Month 24

We first analyzed factors associated with eGFR at ANCA-GN diagnosis in the eGFR cohort (n=80). Estimated GFR was associated with age, percentage of normal and crescentic glomeruli, and IFTA (**Supplementary Table S2**).

**Figure 3:**
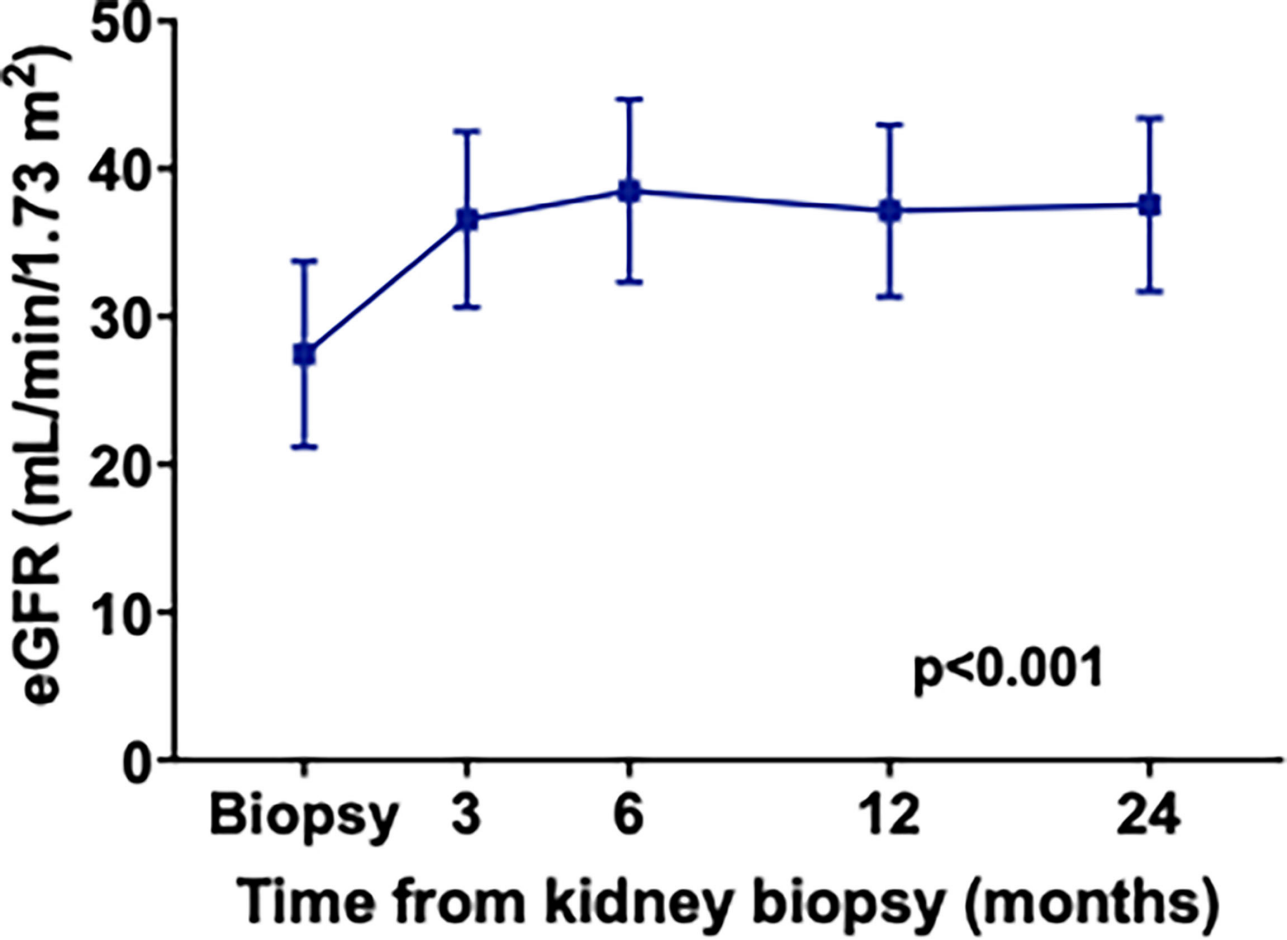
Estimated GFR variation between ANCA-GN diagnosis and month 24 in the eGFR cohort. Statistical analysis was done using a matched one-way ANOVA test.

We next analyzed the eGFR variation over the 2 years following ANCA-GN diagnosis. For this analysis, we used the “eGFR cohort” including patients with exhaustive initial and follow-up eGFR data. As shown in **Figure 3**, the eGFR increased significantly between ANCA-GN diagnosis and month 24 (median eGFR, 16.8 ml/min/1.73 m^2^ at diagnosis and 37.7 ml/min/1.73 m^2^ at 2 years of follow-up).

The univariable analysis of factors associated with eGFR variation between ANCA-GN diagnosis and month 24 is reported in **Table 2**. We did not observe any significant association between baseline characteristics and eGFR variation, even if older age and female gender tended to be associated with greater eGFR gain. Furthermore, we did not observe any association between histopathological classification and eGFR variation. Similarly, the percentage of normal, crescentic, or sclerotic glomeruli and of IFTA at renal biopsy were not associated with eGFR variation; the same holds true for RRS value or categories. The only factor significantly associated with eGFR variation was eGFR at kidney biopsy. Each addition of 10 ml/min/1.73m^2^ eGFR at ANCA-GN diagnosis was associated with a net variation of −3.6 ± 0.8 ml/min/1.73m^2^ eGFR at 24 months (p<0.001). In the multivariable analysis including age, gender, and eGFR at ANCA-GN diagnosis, the only significant factor was eGFR at ANCA-GN diagnosis (ß, −4.11 ± 0.7; CI −5.8, −2.4, p<0.001).

**Table 2:** Univariable analysis of factors associated with eGFR variation between ANCA-GN diagnosis and month 24 in the eGFR cohort.

n=80	eGFR variation (ml/min/1.73 m^2^)	*p*
**Baseline characteristics at ANCA-GN diagnosis**		
Gender, male vs. female	+0.0 [−4.0–+16.1] vs. +22.2 [0.0–+34.3]	0.059
Age (per 10-year increase)	ß: +3.1 ± 1.8	0.086
Hypertension, presence vs. absence	+0.0 [−1.5–+24.9] vs. +11.2 [−3.5–+27.9]	0.370
Diabetes mellitus, presence vs. absence	+0.0 [−2.8–+12.7] vs. +10.9 [−1.0–+30.0]	0.356
MPO ANCA or no ANCA vs. PR3 ANCA	+10.9 [−1.0–+30.0] vs. 2.7 [−3.1–+24.4]	1.000
Organ involvement, presence vs. absence		
Cutaneous signs	+5.5 [−15.3–+37.6] vs. +10.5 [−0.9–25.4]	0.960
Ear, nose, throat	+11.1 [0.7–+26.5] vs. +0.0 [−3.5–+30.0]	0.750
Heart	+7.2 [−18.3–+19.2] vs. +10.2 [−0.9–+27.2]	0.695
Digestive	+5.6 [−14.0–+22.6] vs. +10.2 [−1.0–+30.0]	0.626
Lung	+10.2 [0.0–+22.5] vs. +10.2 [−2.3–29.0]	0.567
Neurological	+26.4 [0.0–+27.5] vs. +8.7 [−2.6–+25.0]	0.408
**Kidney biopsy**		
Initial eGFR (per 10 ml/min/1.73 m^2^ increase)	ß: −3.6 ± 0.8	<0.001
AAV GN classification		0.417
Sclerotic (n=13)	+0.0 [0.0–+17.3]	–
Mixed (n=33)	+10.9 [−7.5–+24.9]	–
Crescentic (n=18)	+13.4 [−0.5–+35.2]	–
Focal (n=16)	+7.0 [−3.9–+36.1]	–
Fibrinoid necrosis, presence versus absence	+10.9 [−1.5–+27.7] vs. +5.5 [−3.5–+25.0]	0.621
IFTA, ≤25% vs. >25%	9.8 [−2.3–+27.9] vs. +9.5 [−1.8–+23.5]	0.468
ATN, presence versus absence	+10.9 [−0.8–+25.0] vs. +0.0 [−8.3–+30.2]	0.309
10% increase in normal glomeruli	ß: −0.9 ± 1.1	0.425
10% increase in crescentic glomeruli	ß: +0.5 ± 1.1	0.649
10% increase in sclerotic glomeruli	ß: −0.1 ± 1.1	0.904
10% increase in IFTA	ß: 0.1 ± 1.1	0.866
Renal risk score, per one-unit increase	ß: +0.64 ± 0.7	0.334
Renal risk score		0.468
Low (n=23)	+7.6 [−8.9–+29.7]	–
Medium (n=37)	+10.9 [−3.1–+24.7]	–
High (n=20)	+2.8 [0.0–26.2]	–
**AAV treatment, use vs. no use**		
Plasma exchange	+19.3 [−3.6–+24.6] vs. +8.7 [−3.7–+24.6]	0.802
Steroid boluses	+10.9 [−3.8–+27.2] vs. −1.0 [−10.1–+14.5]	0.516
Remission induction with cyclophosphamide	+10.5 [−0.8–+27.1] vs. −3.5 [−26.8–+5.3]	0.240
Maintenance regimen with azathioprine	+0.0 [−2.8–+24.9] vs. +10.2 [−2.9–+26.2]	0.604

Results are presented as median eGFR variation with 25–75 percentiles, with + meaning eGFR gain and – meaning eGFR loss.

IFTA, interstitial fibrosis + tubular atrophy; ATN, acute tubular necrosis; GN, glomerulonephritis; AAV, ANCA-associated vasculitis; ANCA, anti-neutrophil cytoplasmic antibodies; GN, glomerulonephritis; MPO, myeloperoxidase; PR3, proteinase-3; eGFR, estimated glomerular filtration rate; IFTA, interstitial fibrosis + tubular atrophy; ATN, acute tubular necrosis.

When the analysis was performed after exclusion of patients that reached ESKD before month 24, female gender and older age were significantly associated with eGFR gain (**Supplementary Table S3**) and initial eGFR. Moreover, RRS tended to be associated with eGFR variation, with each 1-point increase associated with 1.7 ml/min/1.73m^2^ eGFR gain. Use of PE, steroid pulses, and cyclophosphamide as induction–remission regimen were also significantly associated with eGFR gain in the subgroup of patients that did not reach ESKD at 2 years of follow-up.

## Discussion

In the present study, we evaluated the performance of both the histopathological classification of ANCA-GN and of the RRS for ESKD prediction in a large and well-characterized cohort of ANCA-GN patients (20, 21) and studied the factors associated with eGFR at ANCA-GN diagnosis and with eGFR variation at 2 years.

First, we confirmed the value of the RRS for the prediction of ESKD in our cohort, which performed better than the histopathological classification at the end of follow-up. Second, we observed that glomerular lesions and IFTA were associated with eGFR at ANCA-GN diagnosis. Third, we found that eGFR variation at 2 years was mainly dependent on eGFR at ANCA-GN diagnosis in our study. Indeed, we observed that each +10 ml/min/1.73 m^2^ of eGFR at ANCA-GN diagnosis was associated with a net variation of −3.6 ml/min/1.73m^2^ eGFR at 2 years. Interestingly, we were not able to find any association between renal histological lesions at ANCA-GN diagnosis and eGFR variation at 2 years in our study. Moreover, ESKD risk classifications (histopathological classification or RSS) were not associated with eGFR variation at 2 years. Thus, a higher risk of ESKD as predicted at ANCA-GN diagnosis using the histopathological classification or the RRS does not determine the potential of eGFR change at 2 years.

Few studies have compared the prognostic value of the histopathological classification and RRS (22, 23). Using the histopathological classification or RRS, we observed in our cohort survivals similar to those reported in the original studies and subsequent confirmation cohorts (12, 13, 22–34). As some previous studies did, we observed similar prognosis of the mixed and crescentic classes (12, 35). The sclerotic class had an estimated 5-year survival of 51.3%, in line with the original study of Berden et al. and with most subsequent studies (11, 12). In the original study of Brix et al., the renal survival at 3 years was 100%, 84%, and 32% in the low, medium, and high RRS categories (13). In our study, the 3-year survival was in the same ranges (92.2%, 84.6, and 42.3% in low, medium, and high ESKD risk categories, respectively). In addition, in line with previous studies, in the univariable Cox analysis, we observed that medium- and high-risk categories had about a three- and a ninefold increased risk of ESKD, respectively, as compared to low-risk category. In line with other studies, we observed that initial eGFR and percentage of normal glomeruli were significantly associated with ESKD (**Supplementary Figure S3**). However, in contrast to Brix et al. (13), we did not find any association between IFTA and ESKD risk, using the cutoff of 25%.

In line with previous studies, we found that eGFR at diagnosis was negatively correlated with age and percentage of sclerotic glomeruli and positively correlated with percentage of normal glomeruli (14, 15, 36). Initial eGFR was correlated, as expected, with histological classification and RRS. Patients with ATN had lower eGFR at diagnosis than patients without, but the difference was not statistically significant. Interestingly, we observed a significant correlation between the amount of IFTA and initial eGFR. However, the cutoff of 25% was not significantly associated with eGFR at ANCA-GN onset in our cohort.

In the last part of the study, we analyzed the predictors of eGFR variation over 2 years following ANCA-GN diagnosis. Similarly to previous studies, we observed eGFR recovery mainly between diagnosis and month 6 (15, 36). Interestingly, we found that older and female patients tended to gain eGFR, while past studies rather found an inverse correlation with age (14, 15). While we do not have any definite explanation for this observation, we suppose that it may be related to cohort characteristics and/or to the therapeutic management of elderly patients in our centers. Finally, in the multivariable analysis, the eGFR at ANCA-GN diagnosis was the only significant predictor of eGFR variation in our cohort. Interestingly, each +10 ml/min/1.73 m^2^ eGFR at ANCA-GN diagnosis was associated with a net variation of −4.1 ml at 2 years. In other words, and as already observed in previous studies (14, 15), patients with lower eGFR at presentation are also those with greater chances to gain eGFR after ANCA-GN onset.

Interestingly, histological class, glomerular lesions, or IFTA, while being associated with initial eGFR, were not significantly associated with eGFR change. Moreover, a recent study also did not find any association between IFTA and ESKD in ANCA-GN patients using the threshold of 25% for IFTA (27). We suggest that interobserver variations in IFTA quantification may account for these differences. In support, some past studies have shown correlation between IFTA and ESKD, but with higher cutoff of IFTA (14, 37, 38). Given the low interobserver agreement in IFTA quantification in clinical practice (39), we suggest that a simplified RRS limited to eGFR and percentage of normal glomeruli should be considered for ESK prediction. In our study, removing IFTA from the model did not significantly modify ESKD prediction. Finally, the lack of association between histological lesions and eGFR variation may suggest more complex interplay between several histological lesions and non-histological factors.

Our cohort study is limited by its retrospective design and by the fact that therapeutic management has changed over years. Moreover, the nature and duration of treatment in selected subgroups of patients, such as elderly patients, may have been influenced by differences in local clinical practice. Despite these limitations, we observed rates of ESKD consistent with those of other cohorts using the histopathological classification and the RRS. Major strengths of our study are that kidney pathology analysis was centralized and that the cohort is well characterized, allowing to analyze a large number of variables.

In conclusion, this study shows that the RRS predicts ESKD more accurately than the histopathological classification during long-term follow-up, while prediction was not significantly improved at 3 years of follow-up. We also found that eGFR at ANCA-GN onset is associated with glomerular lesions and severity of IFTA, but in our study, these lesions are not major determinants of eGFR change at 2 years. The only determinant of eGFR variation was eGFR at ANCA-GN diagnosis. Interestingly, RRS value was not associated with eGFR change at 2 years, underlining that even patients with a severe disease have a potential for eGFR gain under treatment. Therefore, we suggest cautious interpretation of the RRS in relation to treatment decisions.

## Data Availability Statement

The raw data supporting the conclusions of this article will be made available by the authors, without undue reservation.

## Ethics Statement

The studies involving human participants were reviewed and approved by Comité d’Etique du CHU d’Angers. Written informed consent for participation was not required for this study in accordance with the national legislation and the institutional requirements.

## Author Contributions

BB, JFA, and JFS contributed to conception and design of the study. BB, CB, and PJ organized the database. JFA and JR performed the statistical analysis. BB and PJ wrote the first draft of the manuscript. MCC, CB, JPSA, and AC performed the pathological analysis. JFA and GBP revised the manuscript. NH, SW, AD, CS, JPC, and MC contributed in patient’s care. All authors contributed to the article and approved the submitted version.

## Conflict of Interest

The authors declare that the research was conducted in the absence of any commercial or financial relationships that could be construed as a potential conflict of interest.

## Publisher’s Note

All claims expressed in this article are solely those of the authors and do not necessarily represent those of their affiliated organizations, or those of the publisher, the editors and the reviewers. Any product that may be evaluated in this article, or claim that may be made by its manufacturer, is not guaranteed or endorsed by the publisher.

## Supplementary Material

Supplementary Figure 1 Kidney biopsy assessment in the ESKD risk cohort (n=123). Diagrams show the percentage of patients according to the number of glomeruli per biopsy **(A)**, or percentage of normal **(B)**, crescentic **(C)**, sclerotic **(D)** glomeruli per biopsy or interstitial fibrosis + tubular atrophy (%). **(F)**, percentage of patients according to the Berden classification.

Supplementary Figure 2 Renal Risk Score components according to RRS categories. The diagrams show the percentage of patients according to proportion of normal glomeruli **(A)**, of IFTA **(B)** and of eGFR **(C)** value (<15 mL/min). Comparisons were done using Khi2 test. RRS, renal risk score; IFTA, interstitial fibrosis + tubular atrophy; eGFR, estimated glomerular filtration rate.

Supplementary Figure 3 Forest plot of clinicopathologic parameters associated with ESKD in the ESKD risk cohort. Hazard ratios with their 95% CI and p-values were calculated using univariable cox analysis to predict ESKD. *Renal risk score value between a minimum score of 0 points to a maximum score of 11 points, HR is given for each one point increase; **, per 10% increase in normal glomeruli proportion; ***, per each 10% increase in IFTA; ****, for each 10 ml/min/1.73 m^2^ eGFR increase.

Supplementary Figure 4 ROC curve analysis of ESKD risk at 3-years and end of follow-up using RRS and modified RSS. Area under curve of Renal risk score (red line) and modified RRS (blue line) for ESKD prediction at 3-years **(left panel)** and at the end of follow-up **(right panel)**. p-values indicate AUC comparisons of RRS score and modified RRS. AUC, Area under curve; RRS, renal risk score. Modified RRS was calculated by removing the IFTA score (maximum score of 9). Black p-values indicate AUC comparisons of RRS and modified RRS.

Supplementary Figure 5 Rate of ESKD according to RRS, **(A)** at 3-years and **(B)** at the end of follow-up. RRS categories are indicated with different colours (green, low risk; blue, medium risk; red, high risk).

Supplementary Table 1 Patients characteristics according to Renal Risk Score categories in the ESKD prognosis cohort (n=123).

Supplementary Table 2 Univariable analysis of factors associated with eGFR at ANCA-GN onset in the eGFR cohort. Results are presented as median eGFR or median eGFR variation with 25-75 percentiles. IFTA, interstitial fibrosis + tubular atrophy; ATN, acute tubular necrosis; GN, glomerulonephritis.

Supplementary Table 3 Univariable analysis of factors associated with eGFR variation between ANCA-GN onset and month 24 in the eGFR cohort after exclusion of patients that reached ESKD before month 24 (n=62). Results are presented as median eGFR variation with 25-75 percentiles, with + meaning eGFR gain and – meaning eGFR loss. IFTA, interstitial fibrosis + tubular atrophy; ATN, acute tubular necrosis; GN, glomerulonephritis.
