# Supplementary figures and images for: Assessment of Renal Risk Score and Histopathological Classification for Prediction of End-Stage Kidney Disease and Factors Associated With Change in eGFR After ANCA-Glomerulonephritis Diagnosis
Source: Front Immunol. 2022 Mar 22;13:834878. doi: 10.3389/fimmu.2022.834878 (PMC8981524; doi:10.3389/fimmu.2022.834878)

**A**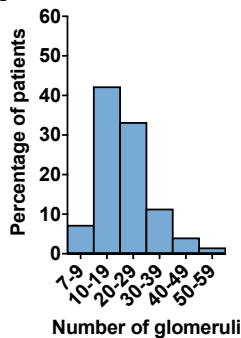**B**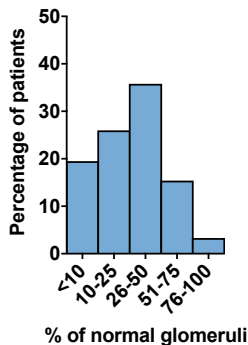**C**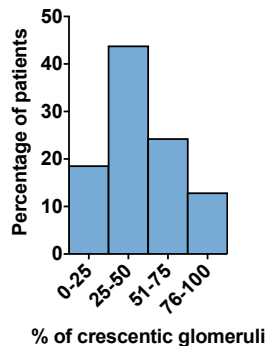**D**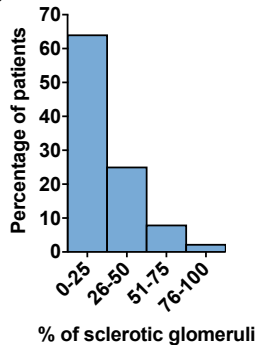**E**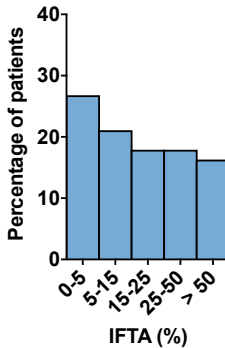**F**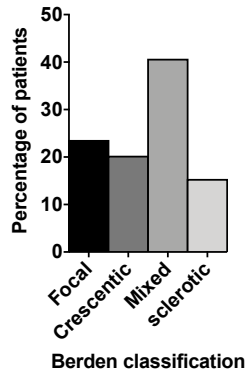

Supplement: Supplementary Figure 1 — Kidney biopsy assessment in the ESKD risk cohort (n=123). Diagrams show the percentage of patients according to the number of glomeruli per biopsy (A), or percentage of normal (B), crescentic (C), sclerotic (D) glomeruli per biopsy or interstitial fibrosis + tubular atrophy (%). (F), percentage of patients according to the Berden classification. [file DataSheet_1.pdf]

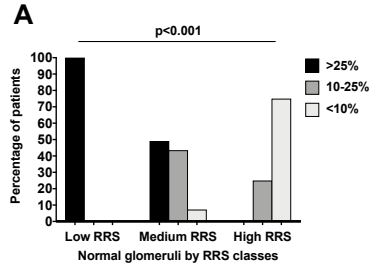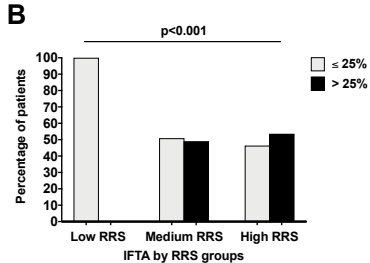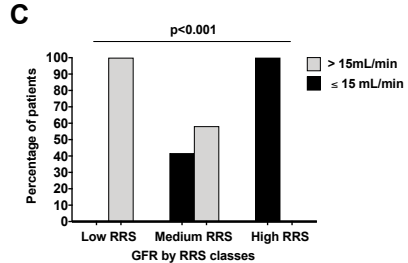

Supplement: Supplementary Figure 2 — Renal Risk Score components according to RRS categories. The diagrams show the percentage of patients according to proportion of normal glomeruli (A), of IFTA (B) and of eGFR (C) value (<15 mL/min). Comparisons were done using Khi2 test. RRS, renal risk score; IFTA, interstitial fibrosis + tubular atrophy; eGFR, estimated glomerular filtration rate. [file DataSheet_2.pdf]

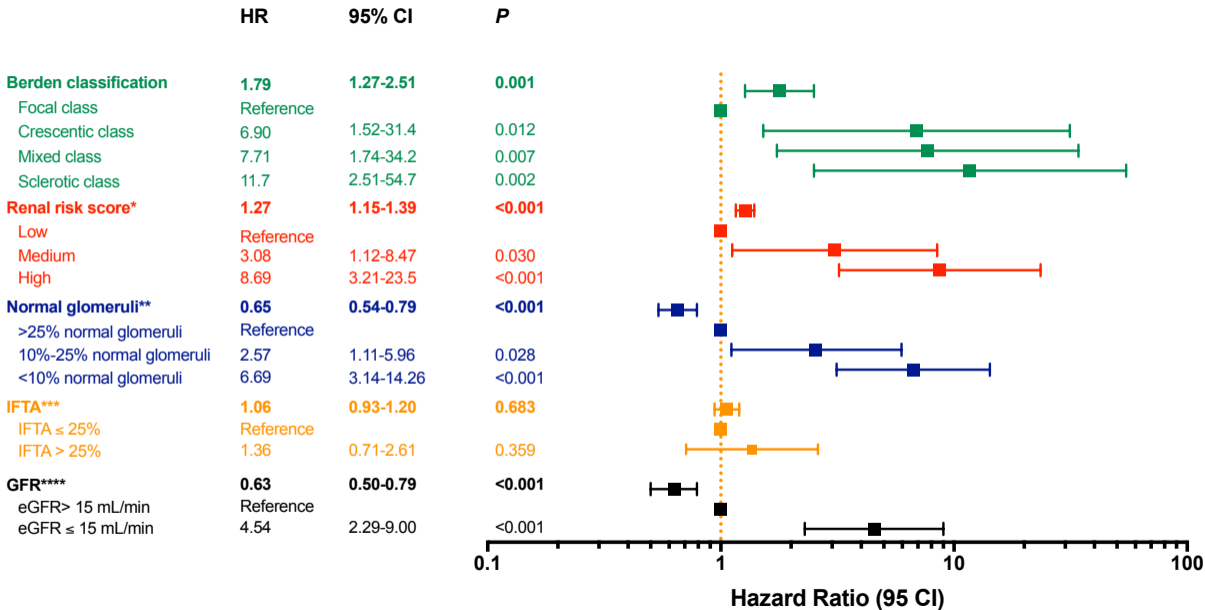

Supplement: Supplementary Figure 3 — Forest plot of clinicopathologic parameters associated with ESKD in the ESKD risk cohort. Hazard ratios with their 95% CI and p-values were calculated using univariable cox analysis to predict ESKD. *Renal risk score value between a minimum score of 0 points to a maximum score of 11 points, HR is given for each one point increase; **, per 10% increase in normal glomeruli proportion; ***, per each 10% increase in IFTA; ****, for each 10 ml/min/1.73 m2 eGFR increase. [file DataSheet_3.pdf]

**ESKD at 3-years**

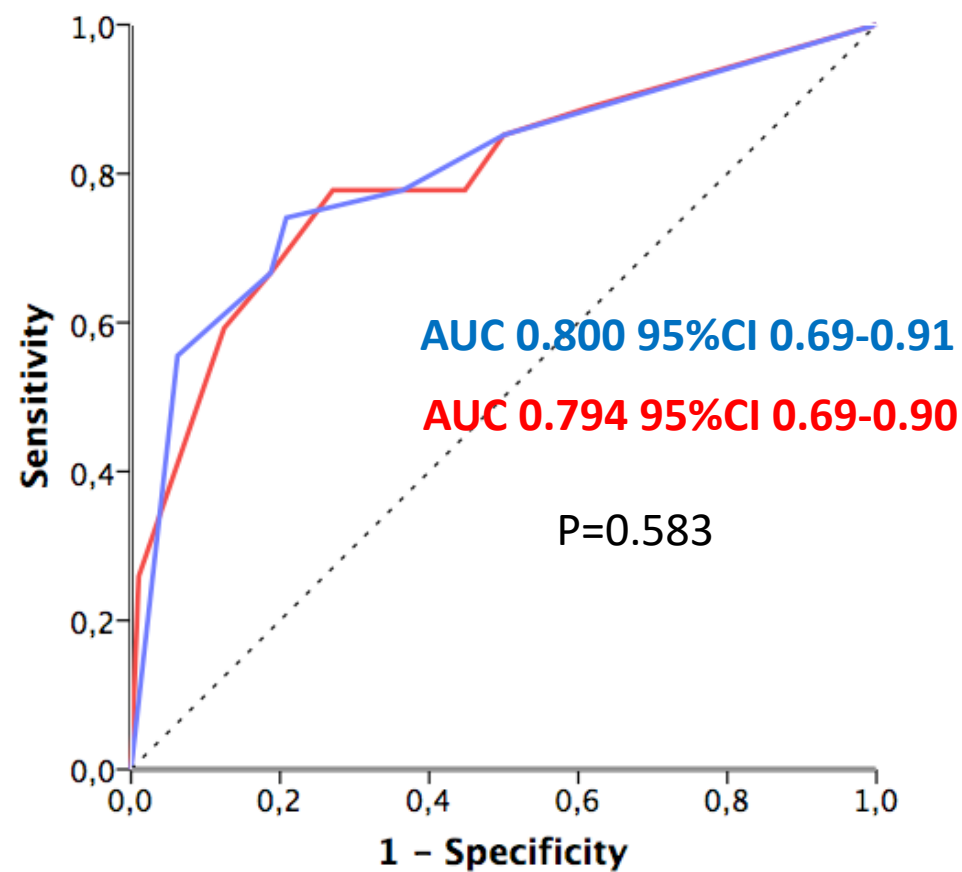

**ESKD at end of follow-up**

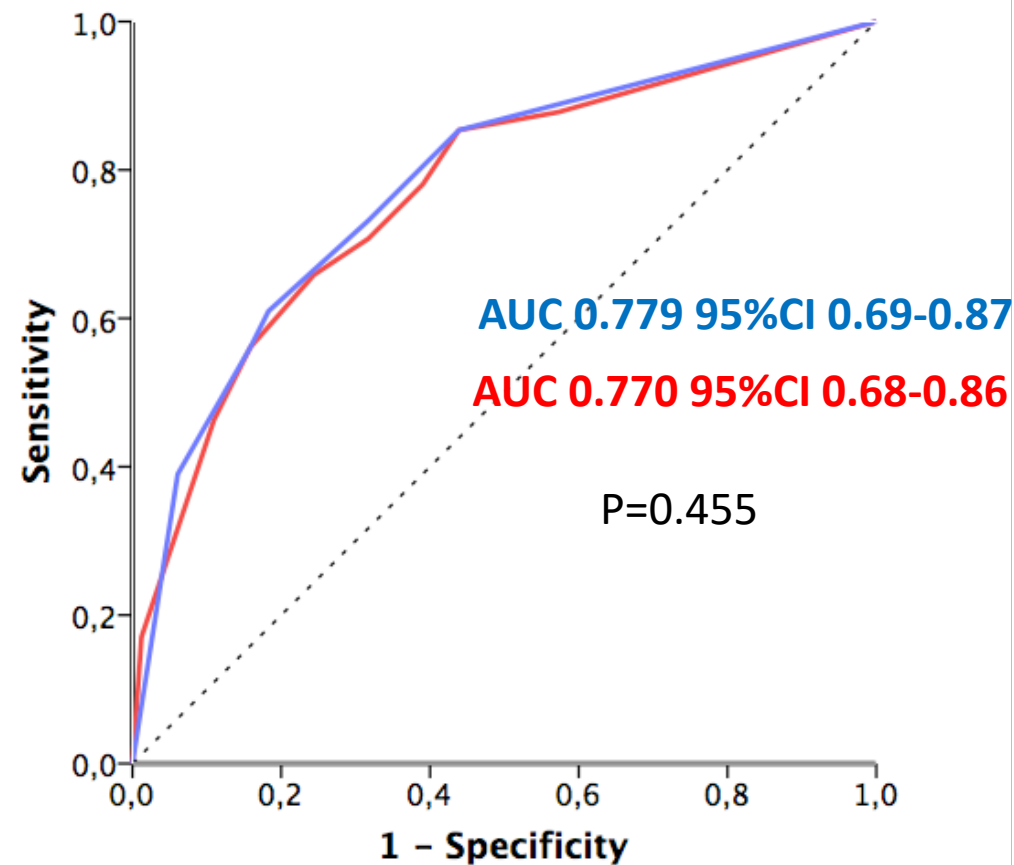

— RRS

— RRS without IFA

Supplement: Supplementary Figure 4 — ROC curve analysis of ESKD risk at 3-years and end of follow-up using RRS and modified RSS. Area under curve of Renal risk score (red line) and modified RRS (blue line) for ESKD prediction at 3-years (left panel) and at the end of follow-up (right panel). p-values indicate AUC comparisons of RRS score and modified RRS. AUC, Area under curve; RRS, renal risk score. Modified RRS was calculated by removing the IFTA score (maximum score of 9). Black p-values indicate AUC comparisons of RRS and modified RRS. [file DataSheet_4.pdf]

**A****3-years follow-up**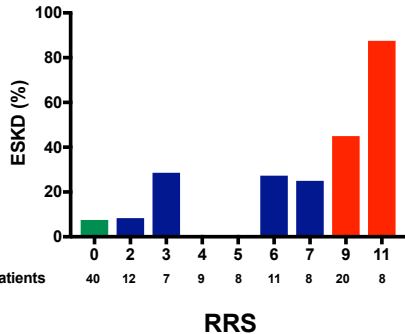

Low RRS  
Medium RRS  
High RRS

**B****End of follow-up**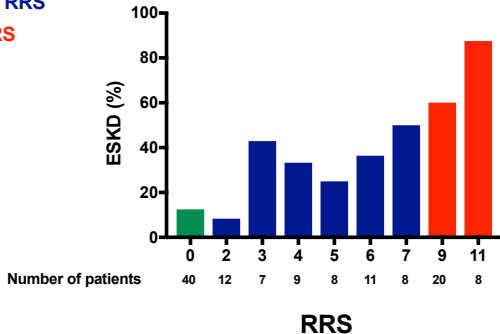

Supplement: Supplementary Figure 5 — Rate of ESKD according to RRS, (A) at 3-years and (B) at the end of follow-up. RRS categories are indicated with different colours (green, low risk; blue, medium risk; red, high risk). [file DataSheet_5.pdf]
