# Supplementary material for: Assessment of Renal Risk Score and Histopathological Classification for Prediction of End-Stage Kidney Disease and Factors Associated With Change in eGFR After ANCA-Glomerulonephritis Diagnosis
Source: Front Immunol. 2022 Mar 22;13:834878. doi: 10.3389/fimmu.2022.834878 (PMC8981524; doi:10.3389/fimmu.2022.834878)
Supplement: Supplementary Table 1 — Patients characteristics according to Renal Risk Score categories in the ESKD prognosis cohort (n=123). [file Table_1.docx]

**Supplemental Table 1. Patients characteristics according to Renal risk score categories in the ESKD prognosis cohort.**

| **RRS categories** | **Low risk** | **Medium risk** | **High risk** |  |
| --- | --- | --- | --- | --- |
|  | **n=40** | **n=55** | **n=28** | **P** |
| **Baseline characteristics at RB** |  |  |  |  |
| Gender, M/F | 22/18 | 34/21 | 20/8 | 0.390 |
| Age, years | 64.5 [53.2-73.7] | 71.0 [62.0-75.0] | 64.5 [53.3-76.3] | 0.072 |
| BMI, Kg/m^2^ | 24.9 [22.8-27.9] | 23.8 [21.9-28.0] | 24.0 [22.4-27.8] | 0.588 |
| Hypertension, n (%) | 18 (45.0) | 33 (60.0) | 13 (46.4) | 0.280 |
| Diabetes mellitus, n (%) | 5 (12.5) | 8 (14.5) | 3 (10.7) | 0.881 |
| **ANCA-associated vasculitis characteristics, n (%)** |  |  |  |  |
| Newly diagnosed | 36 (90.0) | 50 (90.9) | 28 (100.0) | 0.239 |
| BVAS at RB | 17.0 [12.0-21.0] | 15.0 [12.0-19.3] | 15.5 [12.0-20.0] | 0.332 |
| ANCA subtype, n (%) |  |  |  | **0.033** |
| PR3 ANCA | 18 (45.0) | 14 (25.5) | 5 (17.9) | - |
| MPO ANCA or ANCA negative | 22 (55.0) | 41 (74.5) | 23 (82.1) | - |
| Organ involvement at RB |  |  |  |  |
| Cutaneous signs, n (%) | 13 (32.5) | 8 (14.5) | 1 (3.5) | **0.007** |
| Ear, nose, throat, n (%) | 13 (32.5) | 20 (36.4) | 6 (21.4) | 0.355 |
| Heart, n (%) | 2 (5.0) | 4 (7.3) | 1 (3.6) | 0.891 |
| Digestive, n (%) | 2 (5.0) | 3 (5.5) | 1 (3.0) | 1.000 |
| Lung, n (%) | 12 (30.0) | 21 (38.2) | 13 (46.4) | 0.381 |
| Neurological, n (%) | 6 (15.0) | 5 (9.1) | 4 (25.0) | 0.639 |
| Renal |  |  |  |  |
| eGFR, mL/min/1.73 m^2^ | 47.5 [28.6-79.5] | 16.9 [12.8-36.3] | 5.7 [5.0-9.3] | **<0.001** |
| Proteinuria, g/g | 0.58 [0.29-1.09] | 1.16 [0.75-1.86] | 1.73 [1.30-2.51] | **<0.001** |
| Need for renal replacement therapy, n (%) | 2 (5.0) | 10 (18.2) | 16 (61.5) | **<0.001** |
| **Outcomes**, n (%) |  |  |  |  |
| End-stage renal disease | 5 (12.5) | 17 (30.9) | 19 (67.9) | **<0.001** |
| Death | 5 (12.5) | 22 (40.0) | 9 (32.1) | **0.014** |
| **Follow-up** (months) | 42.9 [20.1-107.0] | 26.1 [8.5-69.0] | 13.3 [0.18-52.2] | 0.270 |
