# Supplementary material for: Assessment of Renal Risk Score and Histopathological Classification for Prediction of End-Stage Kidney Disease and Factors Associated With Change in eGFR After ANCA-Glomerulonephritis Diagnosis
Source: Front Immunol. 2022 Mar 22;13:834878. doi: 10.3389/fimmu.2022.834878 (PMC8981524; doi:10.3389/fimmu.2022.834878)
Supplement: Supplementary Table 2 — Univariable analysis of factors associated with eGFR at ANCA-GN onset in the eGFR cohort. Results are presented as median eGFR or median eGFR variation with 25-75 percentiles. IFTA, interstitial fibrosis + tubular atrophy; ATN, acute tubular necrosis; GN, glomerulonephritis. [file Table_2.docx]

**Table 2.**

| **n=80** | **GFR (mL/min/1.73 m^2^)** | ***P*** |
| --- | --- | --- |
| **Baseline characteristics at ANCA-GN diagnosis** |  |  |
| Gender, males vs females | 17.0 [8.2-28.2] vs 17.8 [5-38.6] | 1.000 |
| Age (per 10 year increase) | ß : -9.5 ± 2.1 | **<0.001** |
| Hypertension, presence vs absence | 13.3 [5.0-25.2] vs 20.4 [9.5-40.4] | 0.073 |
| Diabetes mellitus, presence vs absence | 7.2 [5.0-46.9] vs 13.3 [7.2-36.3] | 0.356 |
| MPO ANCA or no ANCA vs PR3 ANCA | 15.2 [6.7-31.7] vs 24.8 [6.1-75.5] | 0.570 |
| Organ involvement, presence vs absence |  |  |
| Cutaneous signs | 31.7 [13.7-80.2] vs 14.0 [6.0-31.7] | 0.061 |
| Ear, nose, throat | 17.2 [7.5-45.1] vs 16.8 [6.4-31.9] | 0.750 |
| Heart | 22.5 [5.0-60.0] vs 16.9 [6.9-34.1] | 0.695 |
| Digestive | 25.9 [5.0-72.7] vs 16.9 [7.2-36.3] | 0.626 |
| Lung | 8.2 [5.0-40.4] vs 19.4 [12.2-36.6] | 0.099 |
| Neurological | 11.1 [5.0-28.2] vs 17.1 [7.2-37.3] | 0.972 |
| **Kidney biopsy** |  |  |
| AAV GN classification |  | **0.002** |
| Sclerotic (n=13) | 5.0 [5.0-14.5] |  |
| Mixed (n=15) | 20.4 [12.9-36.3] |  |
| Crescentic (n=36) | 13.5 [5.0-25.2] |  |
| Focal (n=16) | 50.4 [20.1-87.2] |  |
| Fibrinoid necrosis, presence versus absence | 17.2 [6.8-37.2] vs 15.2 [6.7-31.6] | 0.621 |
| IFTA, <25% vs >25% | 16.8 [7.8-28.1] vs 16.8 [5.0-47.9] | 0.811 |
| ATN, presence versus absence | 13.7 [5.0-31.6] vs 20.4 [9.6-37.9] | 0.309 |
| 10% increase in normal glomeruli | ß : +7.7 ± 1.2 | **<0.001** |
| 10% increase in crescentic glomeruli | ß : -2.5 ± 1.4 | 0.083 |
| 10% increase in sclerotic glomeruli | ß : -4.7 ± 1.4 | **0.001** |
| Renal risk score, per one unit increase | ß : -5.3 ± 0.7 | **<0.001** |
| Renal risk score |  | **<0.001** |
| Low (n=19) | 54.1 [31.7-89.2] |  |
| Medium (n=43) | 16.8 [10.1-28.0] |  |
| High (n=18) | 5.0 [5.0-6.7] |  |

AAV, ANCA-associated vasculitis; ANCA, anti-neutrophil cytoplasmic antibodies; GN, glomerulonephritis; MPO, myeloperoxidase; PR3, proteinase-3; eGFR, estimated glomerular filtration rate; IFTA, interstitial fibrosis + tubular atrophy; ATN, acute tubular necrosis.
