# Supplementary material for: Assessment of Renal Risk Score and Histopathological Classification for Prediction of End-Stage Kidney Disease and Factors Associated With Change in eGFR After ANCA-Glomerulonephritis Diagnosis
Source: Front Immunol. 2022 Mar 22;13:834878. doi: 10.3389/fimmu.2022.834878 (PMC8981524; doi:10.3389/fimmu.2022.834878)
Supplement: Supplementary Table 3 — Univariable analysis of factors associated with eGFR variation between ANCA-GN onset and month 24 in the eGFR cohort after exclusion of patients that reached ESKD before month 24 (n=62). Results are presented as median eGFR variation with 25-75 percentiles, with + meaning eGFR gain and – meaning eGFR loss. IFTA, interstitial fibrosis + tubular atrophy; ATN, acute tubular necrosis; GN, glomerulonephritis. [file Table_3.docx]

**Supplemental Table 3. Univariable analysis of factors associated with eGFR variation between ANCA-GN diagnosis and month 24 in the eGFR cohort after exclusion of patients that reached ESKD before month 24 (n=62).**

| **n=62** | **eGFR variation (mL/min/1.73 m^2^)** | ***P*** |
| --- | --- | --- |
| **Baseline characteristics at ANCA-GN diagnosis** |  |  |
| Gender, males vs females | +10.9 [-3.5 - +24.9] vs +27.5 [+14.5 - +37.7] | **0.009** |
| Age (per 10 year increase) | ß : +4.5 ± 2.0 | **0.027** |
| Hypertension, presence vs absence | +15.8 [-2.5 - +27.7] vs +14.5 [+3.4 - +30.7] | 0.797 |
| Diabetes mellitus, presence vs absence | +2.9 [-3.5 - +2.9] vs +14.8 [-0.8 - +29.7] | 1.000 |
| MPO ANCA or no ANCA vs PR3 ANCA | +15.1 [+4.9 - +31.3] vs 13.0 [-7.7 - +27.2] | 0.786 |
| Organ involvement, presence vs absence |  |  |
| Cutaneous signs | +10.5 [-18.6 - +33.4] vs +15.4 [+4.0 - +26.1] | 0.712 |
| Ear, nose, throat | +11.1 [+4.1 - +23.7] vs +17.6 [-3.4 - +29.7] | 0.459 |
| Heart | +15.7 [-27.8 - +21.4] vs +14.1 [-0.9 - +28.7] | 0.499 |
| Digestive | +11.2 [-3.7 - +18.7] vs +15.4 [-1.0 - +28.0] | 0.659 |
| Lung | +15.4 [-14.8 - +24.6] vs +12.4 [-1.0 - 29.4] | 0.766 |
| Neurological | +22.7 [+8.5 - +24.5] vs +12.7 [-3.4 - +28.7] | 0.606 |
| **Kidney biopsy** |  |  |
| Initial eGFR (per 10 mL/min/1.73 m^2^ increase) | ß :-4.9 ± 0.7 | **<0.001** |
| AAV GN classification |  | 0.756 |
| Sclerotic (n=8) | +9.8 [+0.8 - +25.9] | - |
| Mixed (n=25) | +15.6 [+0.1 - +27.2] | - |
| Crescentic (n=13) | +16.7 [+11.6 - +40.6] | - |
| Focal (n=16) | +7.0 [-3.9 - +36.1] | - |
| Fibrinoid necrosis, presence versus absence | +14.6 [+1.0 - +30.6] vs +15.5 [-1.0 - +26.4] | 1.000 |
| IFTA, ≤25% vs >25% | +14.7 [-0.8 - +31.3] vs +14.3 [-1.3 - +24.8] | 0.786 |
| ATN, presence versus absence | +15.2 [+4.9 - +28.1] vs +10.6 [-6.0 - +36.5] | 0.772 |
| 10% increase in normal glomeruli | ß : -2.4 ± 1.3 | 0.063 |
| 10% increase in crescentic glomeruli | ß : +1.6 ± 1.3 | 0.200 |
| 10% increase in sclerotic glomeruli | ß : +0.2 ± 1.4 | 0.882 |
| 10% increase in IFTA | ß : -0.5 ± 1.2 | 0.652 |
| Renal risk score, per unit increase | ß : +1.7 ± 0.8 | **0.046** |
| Renal risk score |  | 0.115 |
| Low (n=21) | +11.2 [-3.8 - +30.5] |  |
| Medium (n=31) | +13.2 [-0.8 - +26.4] |  |
| High (n=10) | +25.8 [+16.7 - +31.8] |  |
| **AAV Treatment, use vs no use** |  |  |
| Plasma exchange | +26.4 [+20.6 - +39.1] vs +11.5 [-2.9 - +27.4] | **0.006** |
| Steroid boluses | +18.2 [+7.6 - +31.3] vs -1.0 [-10.9 - +14.5] | **0.046** |
| Remission induction with cyclophosphamide | +16.1 [+3.6 - +30.9] vs -3.5 [-26.8 - +5.3] | **0.043** |
| Maintenance regimen with azathioprine | +13.1 [-0.8 - +31.3] vs +15.1 [-0.8 - +27.8] | 0.358 |
